# MRI in Diagnosis of a Giant Prostatic Utricle

**DOI:** 10.1155/2014/217563

**Published:** 2014-07-15

**Authors:** Dustin Johnson, Kushal Parikh, William Schey, Winnie Mar

**Affiliations:** ^1^Department of Radiology, University of Illinois Hospital, 1740 W Taylor Street, Chicago, IL 60612, USA; ^2^Department of Radiology, University of Michigan Hospital, 1500 E. Medical Center Drive, Ann Arbor, MI 48109, USA

## Abstract

A prostatic utricle cyst is an epithelial lined diverticulum arising from the prostatic urethra and usually asymptomatic when small. When enlarged, it may be symptomatic and is typically accompanied by hypospadias. We present a case of a markedly enlarged prostatic utricle in a neonate without hypospadias, demonstrated on voiding cystourethrography (VCUG), ultrasound, and 1.5 Tesla MRI.

## 1. Introduction

Prostatic utricle cysts are believed to represent the incomplete regression of Müllerian ducts. They are commonly diagnosed during childhood as there is an association with hypospadias, pseudohermaphroditism/intersex disorders, and cryptorchidism. Prostatic utricles are often incidentally found on VCUG or sonography. The case presented is unique in that (1) the prostatic utricle was markedly enlarged, (2) detection has seldom been made with MRI, and (3) the presence of an enlarged prostatic utricle was without associated hypospadias.

## 2. Case Report

Prenatal ultrasound revealed a large cystic mass adjacent to the urinary bladder. Upon the initial neonatal exam, in this term baby, a 3-4 cm firm intra-abdominal mass was palpated in the umbilical region. Also, ambiguous genitalia were observed; specifically, no testes were palpated and a micropenis was seen. Subsequent karyotype revealed 46, XY.

The initial postnatal ultrasound demonstrated moderate right hydronephrosis and a distended urinary bladder. No uterus was identified, but directed ultrasound failed to visualize testicles within the scrotum. Furthermore, the previously identified pelvic cystic mass was not visualized.

**Figure 1:**
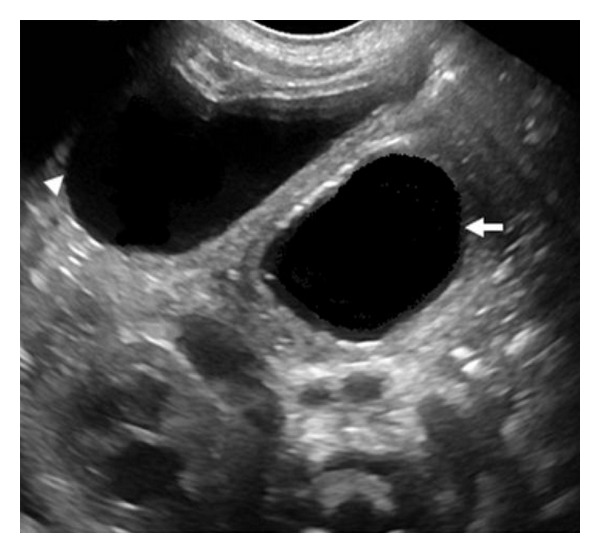
Transverse ultrasound of the pelvis shows a large utricular cyst in the midline (white arrow), posterior to the bladder (white arrowhead).

Decreased urine output over the following two days prompted a repeat ultrasound examination. This study showed two large anechoic cystic structures within the pelvis. One of these structures was identified as the urinary bladder. The second cystic mass was larger and located posterior to the urinary bladder, displacing it anteriorly and toward the right (Figure 1).

**Figure 2:**
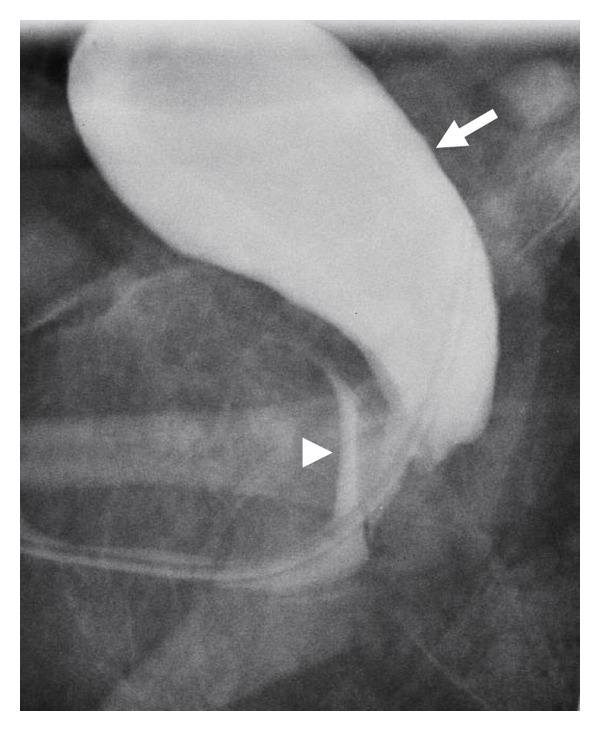
Lateral VCUG demonstrating catheterization of the giant prostatic utricle (white arrow) with retrograde opacification of the urinary bladder (white arrowhead).

VCUG demonstrated a contrast-filled saccular structure. This structure was initially thought to represent the urinary bladder. However, closer inspection revealed that this structure was posterior to the bladder. Since the catheter terminated within this cystic structure, it confirmed its direct communication with the urethra, thus representing a prostatic utricle cyst (Figure 2).

**Figure 3:**
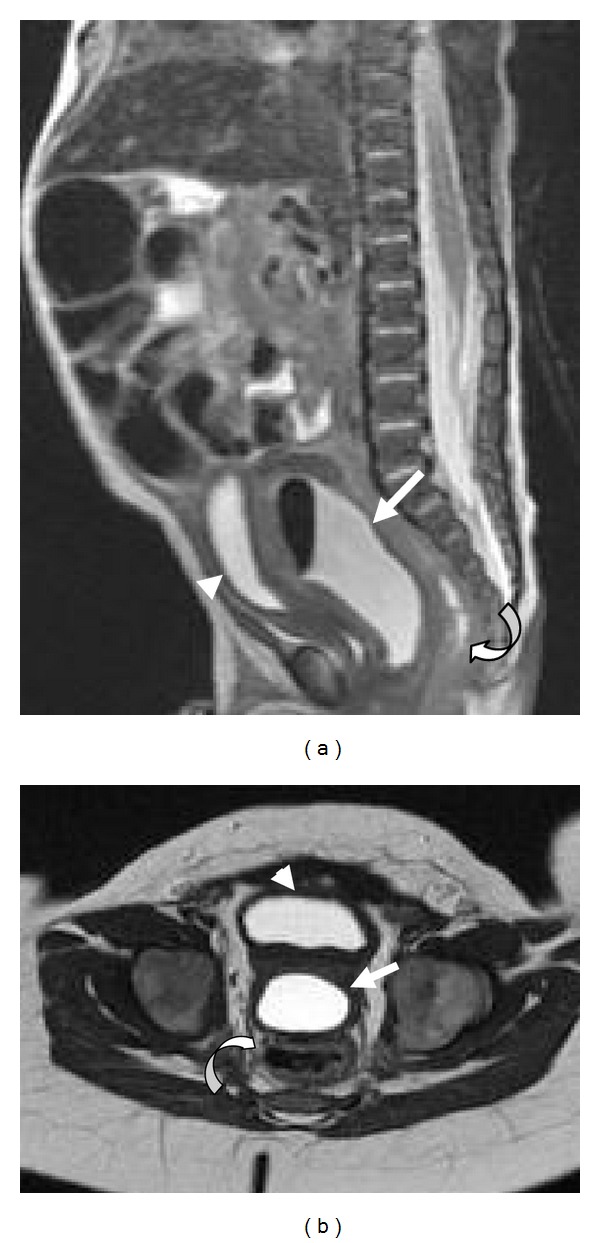
Sagittal (a) and axial (b) T2 turbo spin echo (TSE) MR image show a T2 hyperintense cystic structure representing the prostatic utricle cyst (white arrow), encapsulated by a rim of T2 isointense prostatic tissue, located between the rectum (curved arrow) and urinary bladder (arrowhead).

**Figure 4:**
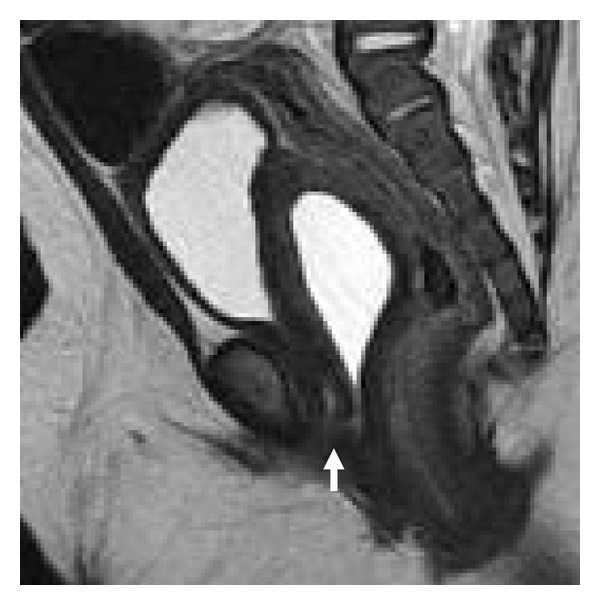
Sagittal T2 TSE MR image revealing the communication between the prostatic utricle cyst and the urethra (white arrow).

MRI at 1.5 T demonstrated a T2 hyperintense, T1 hypointense large cystic structure located between the rectum and urinary bladder, which was encapsulated by a rim of T2 isointense soft-tissue, representing prostatic tissue (Figures 3(a) and 3(b)). This cystic mass directly communicated with the urethra via a thin neck (Figure 4).

Further testing led to the diagnosis of congenital adrenal hyperplasia, for which pharmacologic treatment is in place. Low testosterone was also detected. However, the testes were identified on a 4-month follow-up MRI (not shown), which also showed an interval decrease in size of the prostatic utricle cyst. Definitive treatment of the prostatic utricle was accomplished through complete surgical resection approximately two months later. During this operation, orchiopexy was also performed, which revealed an abnormal appearing right testicle but normal appearing left testicle. The patient's left hydronephrosis resolved after the resection, and the right hydronephrosis improved but persisted to a mild degree. Moreover, follow-up imaging has shown no evidence for residual prostatic utricle and the patient is currently symptom free.

## 3. Discussion

Prostatic utricle cysts are generally believed to represent the incomplete regression of the Müllerian ducts. Others have proposed that they derive from the urogenital system in the prostatic urethra [1]. The most common associated abnormality is hypospadias [2]. Patients presenting with an enlarged prostatic utricle but without hypospadias, on the other hand, are rare. Prostatic utricles are also commonly associated with intersex disorders [3]. Of note, our 46 XY patient exhibited intersex features, most notably in the form of a micropenis, cryptorchidism, and also an abnormal right testicle.

Most prostatic utricular cysts are asymptomatic, especially when small. If large, symptoms typically consist of urinary incontinence, recurrent infections, or stone formation, malignant degeneration has been reported in 3% of prostatic utricles with a peak incidence in the 4th decade of life [4]. Depending on the extent of these accompanying urological abnormalities, patients may remain asymptomatic their entire life or may become symptomatic as older children or adults. On the other hand, as in our case, routine antenatal ultrasound may demonstrate a pelvic cystic mass, which upon further evaluation may reveal a prostatic utricle.

Although initial autopsy-based estimate for prostatic cysts was 1%, the incidence of a giant utricular cyst has not been definitively established [5]. In any case, it is rare for a pelvic cystic mass to reach a size that is detectable antenatally.

The differential for such a pelvic mass in males includes considerations other than a prostatic utricle, such as a bladder diverticulum, urachal cyst, Müllerian duct cyst, or seminal vesicle cyst. As described in this case, giant prostatic utricles communicate with the prostatic urethra and are located midline, posterior to the bladder, and anterior to the rectum. Although they are also midline, Müllerian duct cysts, on the other hand, do not typically communicate with the prostatic urethra. Furthermore, Müllerian duct cysts are frequently encountered during the 3rd to 4th decade of life and are without external genitalia abnormalities [6]. Urachal cysts are clearly distinguished from a prostatic utricle given the former's anterior relationship to the urinary bladder. Seminal vesicle cysts are located lateral to midline and are usually unilateral [6].

With regards to utricular cyst treatment, aspiration/puncture of the cyst has met only moderate success, with approximately half of the cases showing improvement without recurrence. Given these limitations, surgical excision is considered the treatment of choice. Various surgical approaches exist, including suprapubic, midline transvesical, and perineal. Endoscopic procedures improved or cured 82% of the patients in one series [7].

Of note, MRI has been used only rarely to diagnose an enlarged prostatic utricle. To our knowledge, most of these MR evaluations have been reported in older children [8]. Although ultrasound is the first line examination, MRI can be helpful to evaluate a cystic pelvic mass since it can provide improved soft-tissue contrast. Furthermore, MRI allows multiplanar imaging that offers excellent spatial relationships between the cystic mass and adjacent organs, such as the urinary bladder, which may further clarify the diagnosis.

We have presented an unusual case of a neonate with an abnormally large prostatic utricle and an intersex disorder that was not associated with hypospadias, in which MRI was instrumental in the diagnosis. In a patient presenting with a large pelvic cystic mass perinatally with accompanying urological abnormalities, a prostatic utricle cyst should be included in the differential diagnosis.
